# Muscle Synergies During Repetitive Stoop Lifting With a Bioelectrically-Controlled Lumbar Support Exoskeleton

**DOI:** 10.3389/fnhum.2019.00142

**Published:** 2019-04-30

**Authors:** Chun Kwang Tan, Hideki Kadone, Kousei Miura, Tetsuya Abe, Masao Koda, Masashi Yamazaki, Yoshiyuki Sankai, Kenji Suzuki

**Affiliations:** ^1^Artificial Intelligence Laboratory, University of Tsukuba, Tsukuba, Japan; ^2^Center for Innovative Medicine and Engineering, University of Tsukuba Hospital, Tsukuba, Japan; ^3^Department of Orthopaedic Surgery, Faculty of Medicine, University of Tsukuba, Tsukuba, Japan; ^4^Center for Cybernics Research, University of Tsukuba, Tsukuba, Japan; ^5^Faculty of Engineering, University of Tsukuba, Tsukuba, Japan

**Keywords:** exoskeleton, lumbar support, hybrid assistive limb, muscle synergies, bioelectric control

## Abstract

Lower back problems are common in the world, which leads to the development of various lumbar support exoskeletons to alleviate this problem. In general, previous studies evaluating lumbar support devices quantified assistance by reporting the reduction in back muscle activity and perceived fatigue. However, despite the beneficial effects of such devices, the effects of using such exoskeletons on muscle coordination are not well-studied. In this study, we examined the short-term change in muscle coordination of subjects using a bioelectrically-controlled lumbar support exoskeleton in a fatiguing stoop lifting task with muscle synergy analysis. Results indicate that muscle coordination changes were dominated by changes in timing coefficients, with minimal change in muscle synergy vectors. Analysis on muscle coordination changes would be useful to design future generations of exoskeletons.

## 1. Introduction

Lower back problems are health issues encountered throughout the world, as the number of individuals with lower back pain globally is expected to increase substantially (Hoy et al., 2012). The cost and burden of lower back pain on the healthcare system appear to be quite substantial, even when accounting for the different perspectives in various countries (Dagenais et al., 2008). Also when indirect costs, such as reduced productivity and absenteeism from work, are considered, the total cost of lower back pain becomes quite large. Indeed, even healthcare workers like nurses are affected and identified to be the most vulnerable to lower back pain (Yassi and Lockhart, 2013).

The prevalence of this health problem has led to the development of various assistive exoskeletons for industrial purposes (de Looze et al., 2016). They found that such devices are shown to reduce back muscle activity during physical loading. Other versions of such devices have also been developed for general use and caregivers, like the Hybrid Assistive Limb (HAL) for Lumbar Support (Hara and Sankai, 2010) and Smart Suit Lite (Imamura et al., 2011).

With such technology being available, problems like lower back pain in humans can be tackled. A study with a lumbar support exoskeleton showed that human task performance can be enhanced in a snow-shoveling task, while reducing fatigue on the wearer (Miura et al., 2018). In another similar study, Imamura et al. (2017) looked into the short term effects of a soft lumbar support exoskeleton, called the Smart Suit Lite (SSL) (Imamura et al., 2011), on muscular strength of nurses who used the exoskeleton. Their study was motivated by the possibility that long-term users of exoskeletons could experience a decrease in muscular strength. They monitored nurses over a 4-week period, where nurses spent 2 weeks wearing the SSL. At the conclusion of the study, they found a significant decrease in perceived fatigue in the lower back muscles, but with no significant decrease in muscular strength of the nurses, suggesting that the exoskeleton supports task performance without interfering with the wearer.

However, despite recent studies showing task enhancements to human performance when using lumbar support exoskeletons, the effects of such devices on muscle coordination is not fully studied. In this paper, we are interested in examining the effects of lumbar support exoskeletons on the human body from the perspective of muscle coordination. Muscle coordination has been proposed to be modular in nature (d'Avella et al., 2003; Ivanenko et al., 2005). Such modules are known as muscle synergies and it is hypothesized that the central nervous system modulates the activation of muscle synergies to achieve movement. Some studies, however, point out that there are currently limitations in the understanding of how such modular control is actually carried out by the nervous system (Giszter, 2015). Nevertheless, the muscle synergy framework has been useful tool used to characterize and describe a wide range of human and animal movements, as shown in studies like those by Torres-Oviedo et al. (2006), Gizzi et al. (2011), Cheung et al. (2012), Chvatal and Ting (2013), and Cappellini et al. (2016).

Recent related research indicated that exoskeletons indeed change human muscle activation patterns in a locomotion task (Hidler and Wall, 2005; Sylos-Labini et al., 2014; Steele et al., 2017). However, these studies only focus on the joints that are actuated by the exoskeleton. We hypothesize that there might be muscle coordination changes in other parts of the body when using lumbar support exoskeletons, as wearing an exoskeleton is, in fact, putting a foreign object on the human body, leading to an immediate structural change in the peripheral motor system. This structural change could cause difference in muscle coordination in other parts of the body unsupported by the exoskeleton. To observe the muscle coordination change, we use muscle synergy analysis to evaluate possible changes in muscle coordination when a lumbar support exoskeleton is used. This allows us to describe coordination changes with low dimensional metrics, in the form of muscle synergy vectors and timing coefficients. We study the effects of the lumbar support exoskeleton (HAL Lumbar support) on subjects in a box lifting task. In order to differentiate the assistive effects of the exoskeleton, we adopted a fatiguing protocol to exhaust the subjects.

## 2. Methods

This study was carried out in accordance with the recommendations of the University Guidelines for Clinical Trials, Institutional Review Board of University of Tsukuba Hospital, with written informed consent from all subjects. All subjects gave written informed consent in accordance with the Declaration of Helsinki. The protocol was approved by the Institutional Review Board of University of Tsukuba Hospital. The University Guidelines for Clinical Trials conforms to the ethical principles of the Declaration of Helsinki.

### 2.1. Subjects

Twenty healthy subjects (13 male, 7 female), aged 22−43 (31.5±6.6) were recruited from the University of Tsukuba and University of Tsukuba Hospital. Subjects were screened before the study to ensure they are free from neurological and musculoskeletal disorders. All subjects were right-handed.

### 2.2. Lumbar Assistive Device

**Figure 1:**
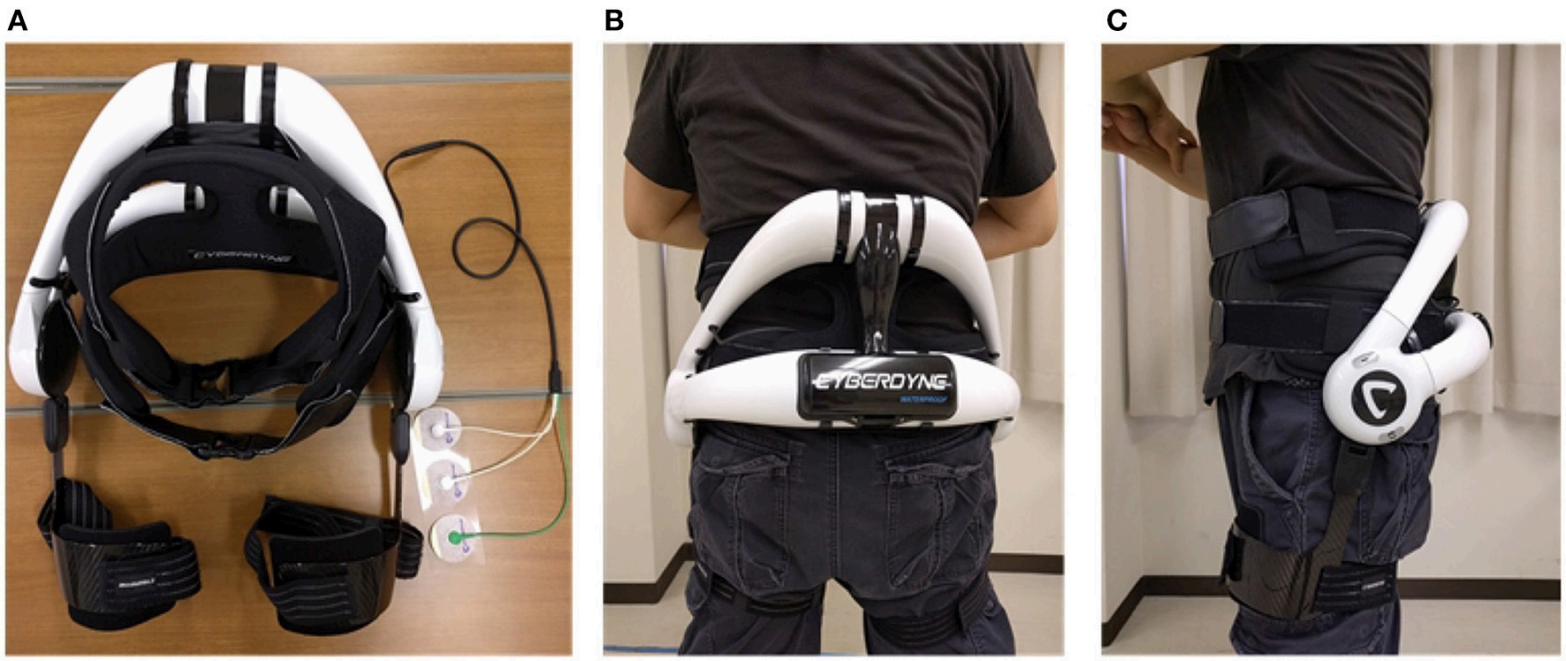
Exoskeleton and fitting. Written informed consent has been obtained from subject for images to be published in this study. **(A)** HAL lumbar support exoskeleton. **(B)** Back view of exoskeleton on subject. **(C)** Side view of exoskeleton on subject.

The commercial version of the lumbar support exoskeleton (Figure 1), named HAL for Care Support (Cyberdyne, Ibaraki, Japan) Hara and Sankai (2010), was used in our study. The device consists of a frame and two actuating units attached to its sides. The frame is designed to restrict the movement of the lumbar vertebrae. Absolute angles of the user's trunk are measured with a triaxial accelerometer, and relative joint angles are measured with potentiometers in the actuators. The exoskeleton is fastened to the user with straps and fasteners, which wrap around the user's trunk and thighs. The actuators provide assistive torque about the hips by applying force on the thigh and trunk. The assistive torque is triggered and controlled by muscle activations, measured by electrodes attached to the skin surface above the user's lumbar erector spinae muscles. A gain parameter on the muscle activations is manually adjusted until the user of the exoskeleton feels comfortable.

#### 2.2.1. Electromyography (EMG)

Skin preparation included wiping down the muscle bellies with alcohol swabs. 8 wireless, surface EMG electrodes (Trigno Lab, Delsys Inc., Boston, MA, USA) were placed bilaterally over the muscle bellies of: biceps brachii (BB), latissimus dorsi (LD), erector spinae (ES) and gluetus maximus (GM). EMG data were sampled at 2000 Hz.

#### 2.2.2. Motion Capture System

Motion tracking of subjects was achieved with the Vicon Motion Capture system (MX System, 16 T20S Cameras, VICON, Oxford, UK). 6 reflective markers were placed bilaterally on the acromion, great trochanter and lateral malleolus. Motion tracking was synchronized with EMG and sampled at 100 Hz.

### 2.3. Experiment Protocol

Subjects were asked to perform 2 sessions (one with HAL and one without HAL) of stoop lifting/placing, until they feel they cannot continue. In each session, subjects were asked to lift and place a small box, (for males, 12 kg, for females, 6 kg). A metronome was used to guide the speed of the subject's action. The metronome was set to 30 beats per minute, which approximately allowed the subject to perform either one lift or place action every 2 s. A 15-min break was given after each session to allow the subject to recover before starting the second session. Order of sessions were randomized for each subject (either starting with HAL or starting without HAL) to account for accumulated fatigue. Out of 20 subjects, We have 12 subjects that started the experiment without HAL, and the remaining 8 subjects, started with HAL.

Subjects were given time to familiarize themselves with the task and exoskeleton before each session until they feel ready. A silent observer counted the number of times the subject lifted the box. At the end of each session, subjects were also asked to evaluate their perception of fatigue on a Visual Analog Scale (VAS) from 0 to 10. The scale used is a 10 cm long continuous line, with the left end marked as “0” and the right end marked as “10.” Subjects indicated their perceived fatigue with a mark anywhere on the line. The distance of the mark to “0” was measured and recorded as the perceived fatigue.

### 2.4. Data Analysis

#### 2.4.1. Software

Data extraction, NNMF and the rest of the processing were performed with scripts on MATLAB 9.3 (Mathworks, Natick, MA, USA).

#### 2.4.2. Preprocessing

EMG data was first filtered with a 4th order, zero-lag Butterworth band-pass filter at 30–400 Hz. The bandpassed EMG was then filtered with a Hampel filter, with the parameters, time window, win = 200 and a threshold of σ = 4 (standard deviations), to remove artifacts. Finally, EMG data was fully rectified and low-passed with a 4th order, zero-lag Butterworth low-pass filter at 6 Hz to obtain the EMG envelope.

#### 2.4.3. Extraction of EMG Based on Kinematic Data

**Figure 2:**
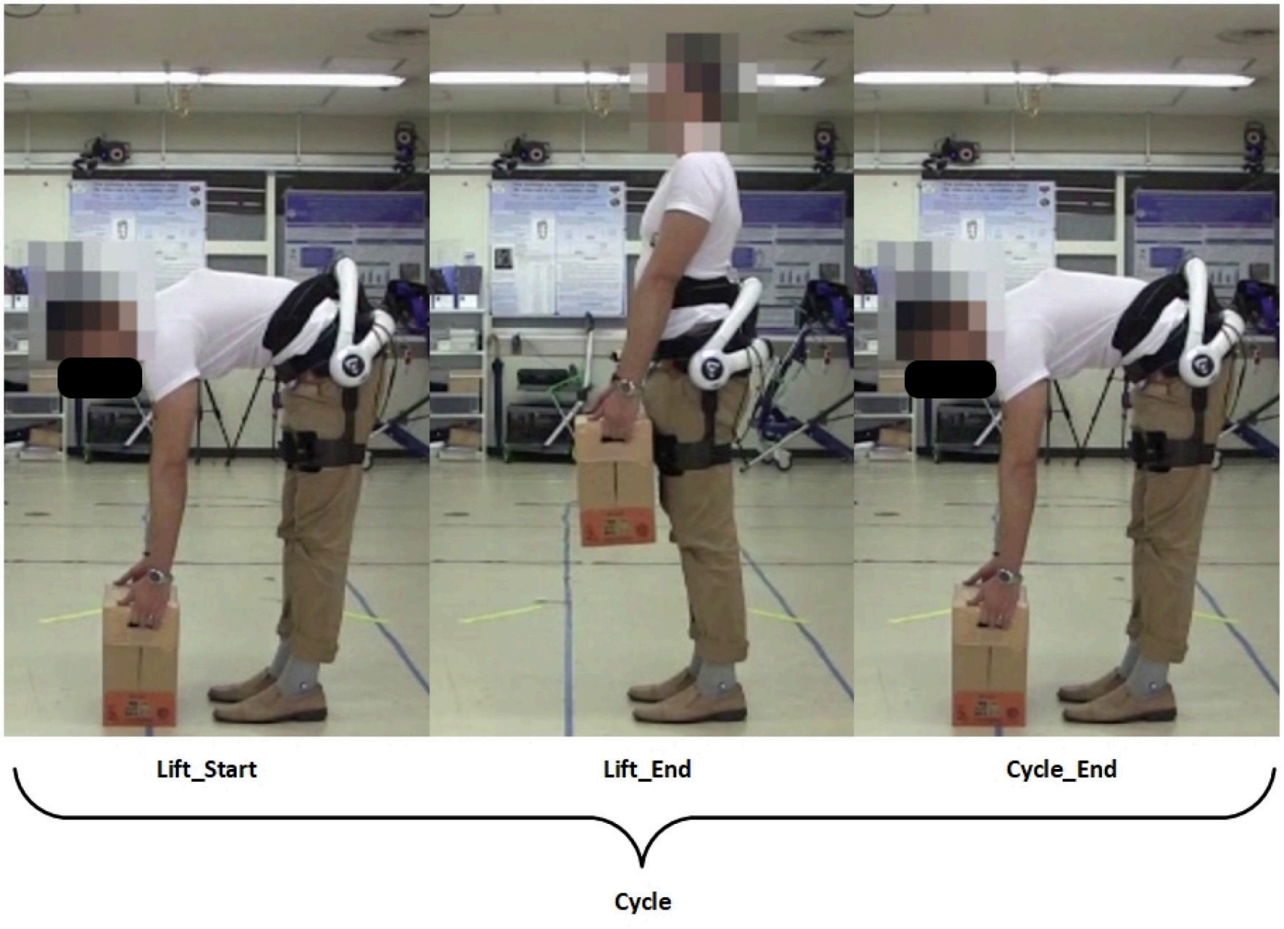
Definition of a lifting cycle. Written informed consent has been obtained from subject for images to be published in this study.

A lifting cycle consist of the subject lifting the box to an upright position, and placing the box back down again, as shown in the figure below (Figure 2).

Four conditions were defined for analysis. They are:

1. No HAL Non-Fatigue
2. No HAL Fatigue
3. HAL Non-Fatigue
4. HAL Fatigue

The “Non-Fatigue” condition is defined to be 3 consecutive and consistent lifting cycles within the first 20% of the total number of lifting cycles for the session. This is to account for adaptation of subjects to the task. The last 3 consecutive lifting cycles for each session were defined as the “Fatigue” condition.

From the synchronized tracks of EMG and motion data, the EMG envelope of 3 consecutive stoop lifting cycles, with consistent movement, were extracted. Each extracted cycle was normalized by its standard deviation and also time-normalized to 100 time points. Finally, the extracted envelopes were concatenated to obtain a 300-by-8 matrix.

**Figure 3:**
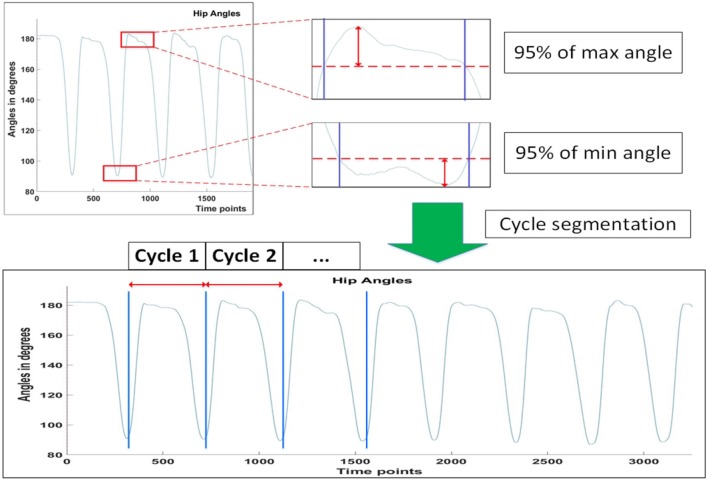
Thresholding of angle values and cycle segmentation of subjects.

Consistency in peak angles and angular velocity were determined for the Non-Fatigue conditions by selecting 3 consecutive lifting cycles with the minimum total absolute difference in peak angles and angular velocities (Equation 1). This is defined as:

(1) $$\begin{array}{r} i_{Non-Fatigue}={\text{argmin}}_{i}\left(\Theta_{i}^{U}+\Theta_{i}^{D}+\Omega_{i}^{U}+\Omega_{i}^{D}\right) \end{array}$$

where the variables are defined as:

(2) $$\begin{array}{r} \Theta_{i}^{U}=|\theta_{i}^{U}-\theta_{i+1}^{U}|+|\theta_{i}^{U}-\theta_{i+2}^{U}|+|\theta_{i+1}^{U}-\theta_{i+2}^{U}| \end{array}$$

(3) $$\begin{array}{r} \Theta_{i}^{D}=|\theta_{i}^{D}-\theta_{i+1}^{D}|+|\theta_{i}^{D}-\theta_{i+2}^{D}|+|\theta_{i+1}^{D}-\theta_{i+2}^{D}| \end{array}$$

(4) $$\begin{array}{r} \Omega_{i}^{U}=|\omega_{i}^{U}-\omega_{i+1}^{U}|+|\omega_{i}^{U}-\omega_{i+2}^{U}|+|\omega_{i+1}^{U}-\omega_{i+2}^{U}| \end{array}$$

(5) $$\begin{array}{r} \Omega_{i}^{D}=|\omega_{i}^{D}-\omega_{i+1}^{D}|+|\omega_{i}^{D}-\omega_{i+2}^{D}|+|\omega_{i+1}^{D}-\omega_{i+2}^{D}| \end{array}$$

where $\theta_{i}^{U},\theta_{i}^{D},\omega_{i}^{U}$ and $\omega_{i}^{D}$ are the *i*th peak angles and angular velocities, respectively, of the hip joint projected to the sagittal plane, during lifting up and down. Θ and Ω are vectors representing the total absolute differences in peak angles and angular velocities. The superscripts represent the phase of the lifting cycle subject is in, with Θ^*U*^ and Ω^*U*^ representing transition from the Lift_Start to Lift_End phrase, and Θ^*D*^ and Ω^*D*^ representing transition from the Lift_End to Cycle_End phrase (Figure 2). Peak angles are additionally defined to be 95% of the actual peak values, so as to account for minute movements of the subjects when they are maintaining stability. Figure 3 depicts how the threshold for hip angle values were applied to segment each lifting cycle.

#### 2.4.4. Task and Kinematics Analysis

Subjects were evaluated on the number of times they were able to lift the box and their perceived fatigue. Peak hip angles and angular velocities of each lifting cycle during each session were also evaluated. Instantaneous velocity profiles were first extracted by differentiation of the vector of hip angle values for each action. The velocity profiles were averaged over 3 actions for each subject and further averaged for all subjects for each condition. The obtained angular velocity profiles were then resampled to 100 time points for plotting. In addition, Root Mean Square (RMS) values of the EMG of each muscle were evaluated for each condition defined in section 2.4.3.

#### 2.4.5. Muscle Synergy Extraction With NNMF

NNMF was then used to extract muscle synergies and timing coefficients from the concatenated EMG data. This was performed with Matlab's NNMF function (Matlab Version 9.3, 2017b), using the multiplicative update algorithm. Parameters for the tolerance for the residual (TolFun) was given as 1*e* − 6 and the tolerance for the relative change in elements (TolX) was given as 1*e* − 4. The algorithm was repeated 300 times with different random starting values of the synergies and timing coefficients. Results with the lowest root mean square residual were taken to be the best. Synergies were allowed to vary during the decomposition process.

The choice of number of synergies were determined with the criteria of when the variance-accounted-for (VAF_*muscle*_) for each muscle vector was above 75% (Torres-Oviedo and Ting, 2007). From our results below (section 3), we fixed the choice of the number of synergies to be 3, as it is sufficient to represent the EMG profiles of all subjects.

The VAF is defined as 100*(uncentered Pearson correlation coefficient) (Torres-Oviedo et al., 2006). This is given as:

(6) $$\begin{array}{r} VAF=100\cdot \left(\frac{{\left(\overset{m}{\underset{j=1}{\sum }}\overset{n}{\underset{i=1}{\sum }}X_{nm}\cdot Y_{nm}\right)}^{2}}{\left(\overset{m}{\underset{j=1}{\sum }}\overset{n}{\underset{i=1}{\sum }}X_{nm}^{2}\right)\cdot \left(\overset{m}{\underset{j=1}{\sum }}\overset{n}{\underset{i=1}{\sum }}Y_{nm}^{2}\right)}\right) \end{array}$$

where *n* is the number of datapoints for each channel, and *m* is the number of channels. For the single muscle vector case, *m* is simply 1. *X*_*nm*_ and *Y*_*nm*_ are the matrices containing the reconstructed and original signal, respectively. VAF calculation code is adapted from the “rsqr_uncentered” function in the file “PosturalData_NMFvsPCA_GUI_July2013” given in Neuromechanics Lab (2016).

**Figure 4:**
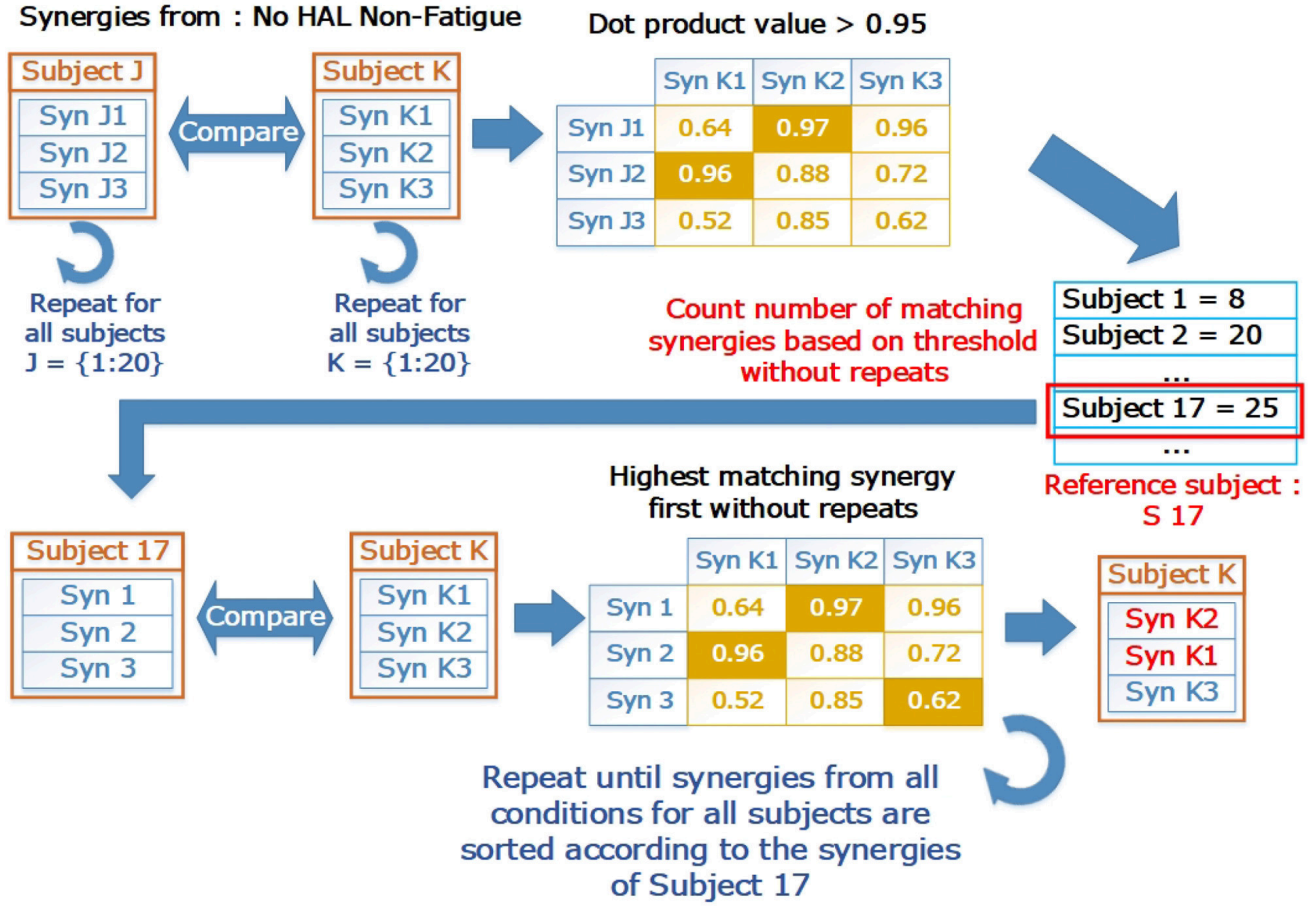
The sorting process in graphical form. The reference subject was selected by counting the number of matching synergies. Letters “J” and “K” refer to loop indices to indicate how the comparison is carried out in a loop to test all pairs of subjects. “J” are the indices for the outer loop, while “K” are the indices for the inner loop. The base condition for the subject (Subject 17, No HAL Non-Fatigue) was then selected as a reference where all other synergies from different conditions and subjects were matched with. After matching, the synergies were sorted according to the indices of the reference subject.

To ensure that the synergies were in the correct order, we sorted the muscle synergy vectors, and their corresponding timing coefficients in relation to a reference subject, using a procedure similar to the greedy search procedure defined in Overduin et al. (2012). We first chose a reference subject by comparing the synergies extracted from the base condition (No HAL Non-fatigue) for each subject. The subject with the highest number of matching synergies to the subject population was selected as the reference. After that, synergies and timing coefficients of all subjects from all the conditions were sorted according to the reference subject. Figure 4 provides a graphical view on the sorting process. Briefly, the sorting procedure compares a reference synergy vector (X, from Subject 17 in our case), with another synergy vector from a different condition/subject (Y), and pairs them such that the dot product value between X and Y is the highest. This pair is then removed from comparison. The procedure is repeated until all synergy vectors are paired. Indices of the vector being sorted (Y) were then arranged to match the order of the reference vector.

#### 2.4.6. Synergy Analysis

We first evaluated the reconstruction quality with the VAF for each condition. This is for deciding the number of synergies used for further analysis. As mentioned in the section above (section 2.4.5), the reconstruction quality is considered sufficient when the VAF for all individual muscle vectors are above 75%. The use of the uncentered correlation coefficient is due to that it is proposed to be more stringent than the classic centered correlation coefficient, as it evaluates both the shape and magnitude of the data (Torres-Oviedo et al., 2006).

We also evaluated the magnitude of change when using Lumbar HAL by evaluating the similarity between sets of conditions with the centered Pearson correlation coefficient (*R*). Since we are interested in the overall difference, the centered correlation coefficient would be sufficient. Muscle synergy vector comparison with the scalar dot product (Cheung et al., 2012) was also performed. This is to evaluate the difference in contents of the muscle synergy vectors, as the calculating the metric normalizes each vector prior to comparison. Each muscle synergy vector was compared with the corresponding vector in the same position for different condition. Timing coefficients were compared with the Uncentered Pearson Correlation Coefficient, in a similar manner as the muscle synergy vectors. This is for evaluating the shape and magnitude differences between timing vectors for different conditions.

To evaluate the significance of the change in synergies against the chance level, we extracted synergies from a random dataset. This dataset is generated by shuffling the data in each EMG muscle channel independently. This is done for every subject. Shuffled EMG data were extracted for processing based on the indices chosen in section 2.4.3. The shuffling and extraction were repeated until 100 sets of raw EMG were obtained for all 4 conditions (4 × 100 dataset, each dataset containing 300 datapoints-by-8 channels), for every subject. Preprocessing as described in section 2.4.2 was performed on the extracted data. Synergies and timing coefficients were extracted by NNMF described in section 2.4.5, and compared in a similar way as the paragraph above (section 2.4.6). Synergies with the highest similarity value from each of the 4 conditions (Best 1 out of 100) were chosen to be the chance level for comparison.

Similar to Jacobs et al. (2018), to evaluate the amount of mutual information between different conditions, synergy weights from different conditions were held fixed while the timing coefficients were optimized again with the a modified NNMF algorithm. The parameters for this algorithm is as described in section 2.4.5 (Multiplicative update rule, TolFun: 1*e* − 6, TolX : 1*e* − 4, Replicates : 300). Evaluation of the timing coefficients were also performed in a similar manner, with the modified algorithm using the same parameters, but with the timing coefficients fixed instead of the synergy weights.

#### 2.4.7. Statistical Analysis

Statistical comparison was performed on paired data with the Wilcoxon Signed-Rank Test. The 2-way ANOVA is used to independently compare the RMS values of muscles under different conditions. Significance was considered in comparisons with *p* < 0.05.

## 3. Results

### 3.1. Task Related Metrics

#### 3.1.1. Number of Task Repetitions and Perceived Fatigue

**Figure 5:**
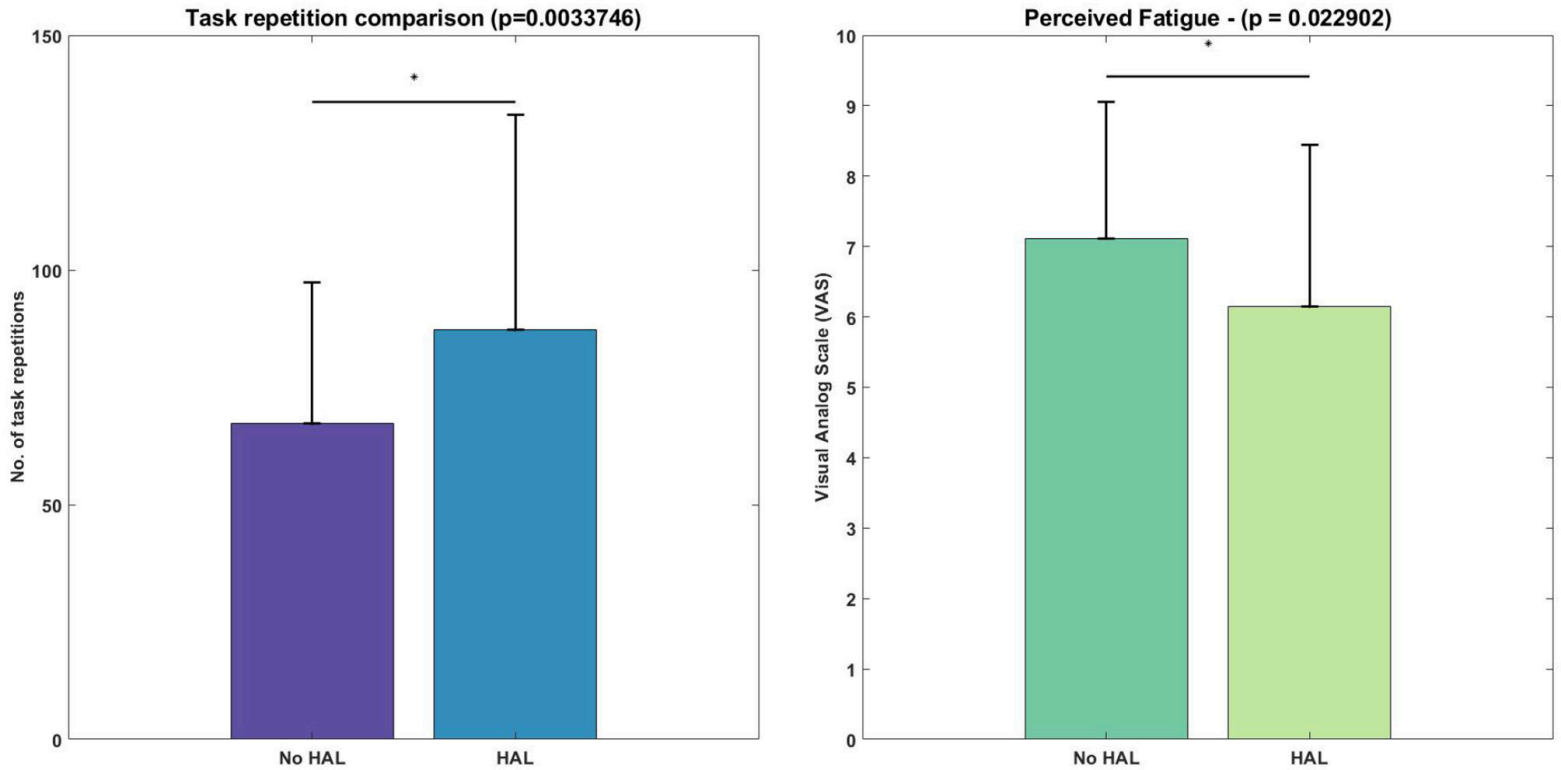
Difference in number of lifting cycles achieved and perceived fatigue, evaluated with the Wilcoxon Signed-Rank test. Asterisks denote significance level of *p* < 0.05.

The figure (Figure 5) below depicts the difference in task repetitions.

Figure 5 depicts the task metrics. Subjects were able to perform significantly more lifting cycles using the exoskeleton, as compared with not using the exoskeleton (HAL condition : 87.2 ± 45.93 vs. No HAL condition: 67.25 ± 30.17, *p* = 0.0034 < 0.05). Perceived fatigue was significantly less when using the exoskeleton as compared to when they were not using the exoskeleton (HAL condition : 6.15 ± 2.30 vs. No HAL condition : 7.12 ± 1.94, *p* = 0.023 < 0.05).

#### 3.1.2. Kinematics

**Figure 6:**
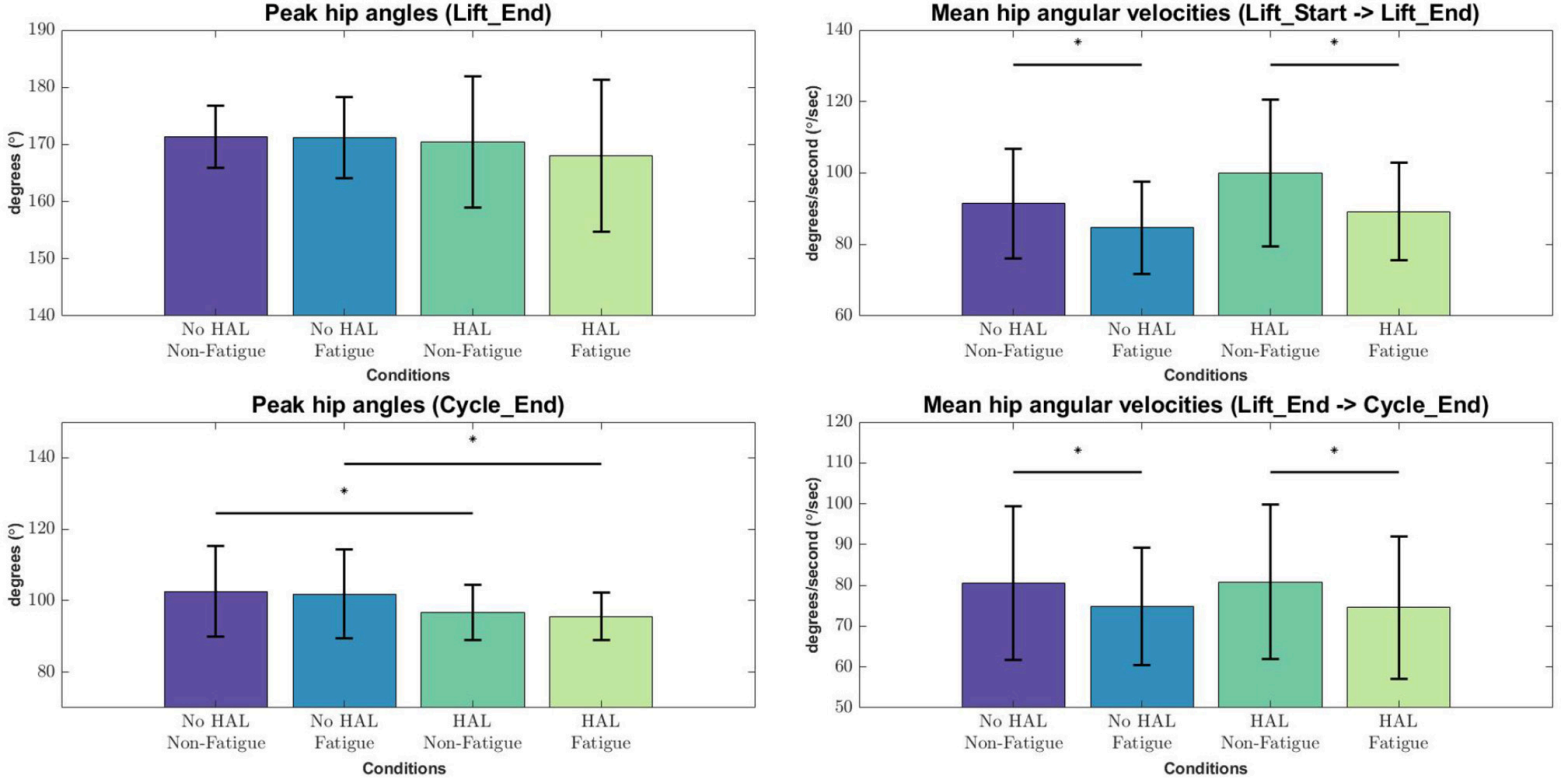
Peak angles and velocities for each condition during each phase of the lifting cycle, evaluated with the Wilcoxon Signed-Rank test. Asterisks denote significance at the level *p* < 0.05.

From the lifting cycle depicted in Figure 2, there were no significant differences in peak hip angles between conditions in the “Lift_End” phase (Figure 6). However, peak hip angles in the “Cycle_End” phase were significantly different when in the Non-Fatigue condition, both with and without HAL (*HAL*: 96.65 ± 7.74 vs. *No HAL* 102.61 ± 12.62, *p* = 0.025 < 0.05). Similarly, significant differences were observed in the peak hip angles in the Fatigue condition, with HAL and without HAL (*HAL* : 95.61 ± 6.68 vs. *No HAL* 101.90 ± 12.37, *p* = 0.019 < 0.05).

**Figure 7:**
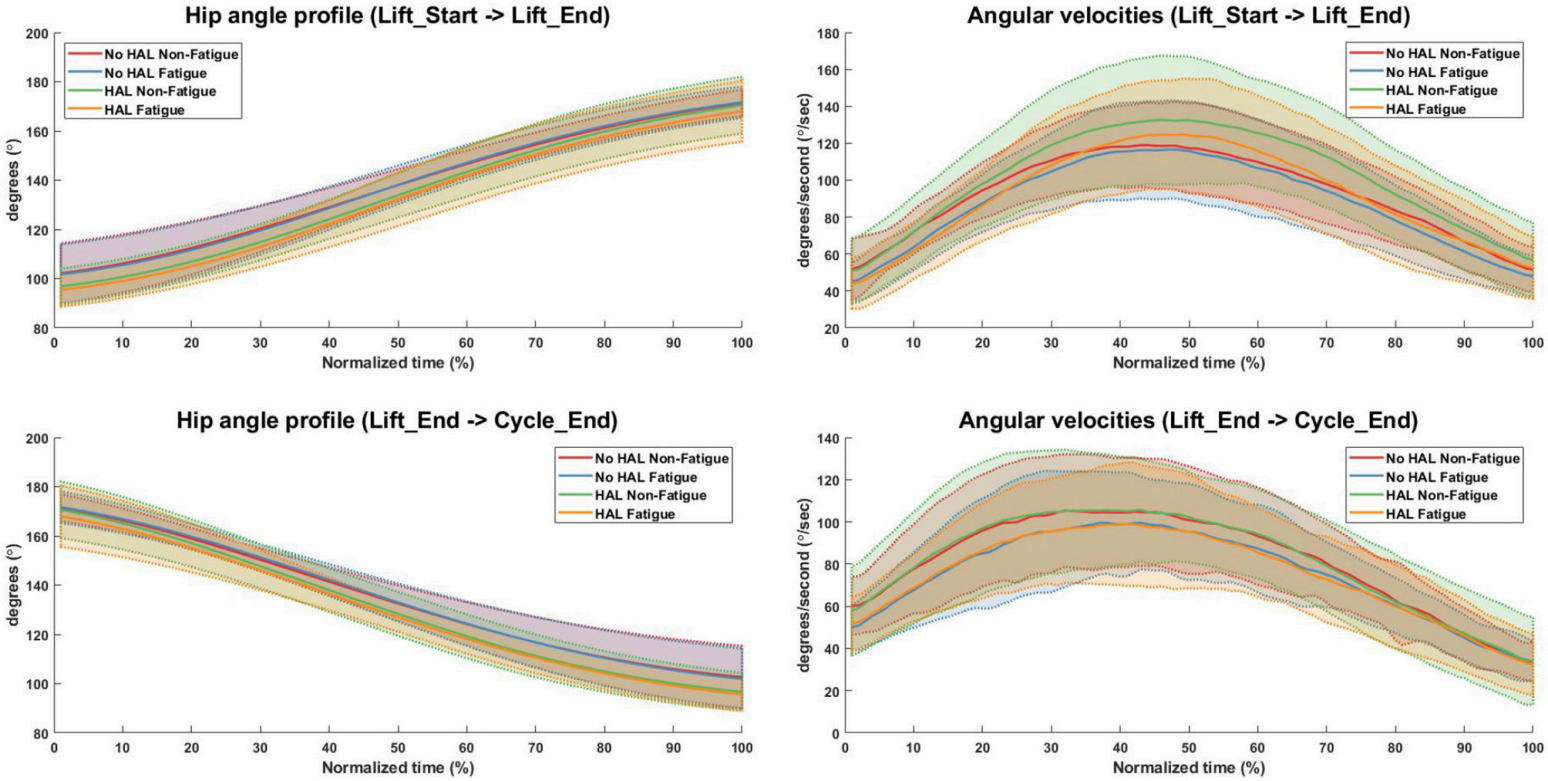
Hip angle and angular velocity profiles for all conditions. Bold lines indicate the mean velocity profile of all subjects, while shaded areas are the standard deviation. Dotted lines at the boundary of the shaded areas are drawn for better visualization.

Angular velocities were significantly different in the “Lift_Start → Lift_End” phase, as subjects appear to slow down as they are fatigued, regardless of the exoskeleton [*No HAL Non-Fatigue*→*No HAL Fatigue* (91.51 ± 15.33 to 84.67 ± 12.84, *p* = 0.0022 < 0.05) and *HAL Non-Fatigue*→*HAL Fatigue* (100.03 ± 20.62 to 89.24 ± 13.56, *p* = 0.00052 < 0.05)]. A similar trend can be also observed in the “Lift_End → Cycle_End” phase, where subjects slow down significantly when they become fatigued, both with and without the exoskeleton [*No HAL Non-Fatigue*→*No HAL Fatigue* (80.60 ± 18.83 to 74.80 ± 14.42, *p* = 0.017 < 0.05) and *HAL Non-Fatigue*→*HAL Fatigue* (80.83 ± 18.92 to 74.55 ± 17.50, *p* = 0.028 < 0.05)]. Figure 7 provides a detailed view of the mean instantaneous velocity profiles of the hip angular velocity under different conditions.

### 3.2. EMG Analysis

**Figure 8:**
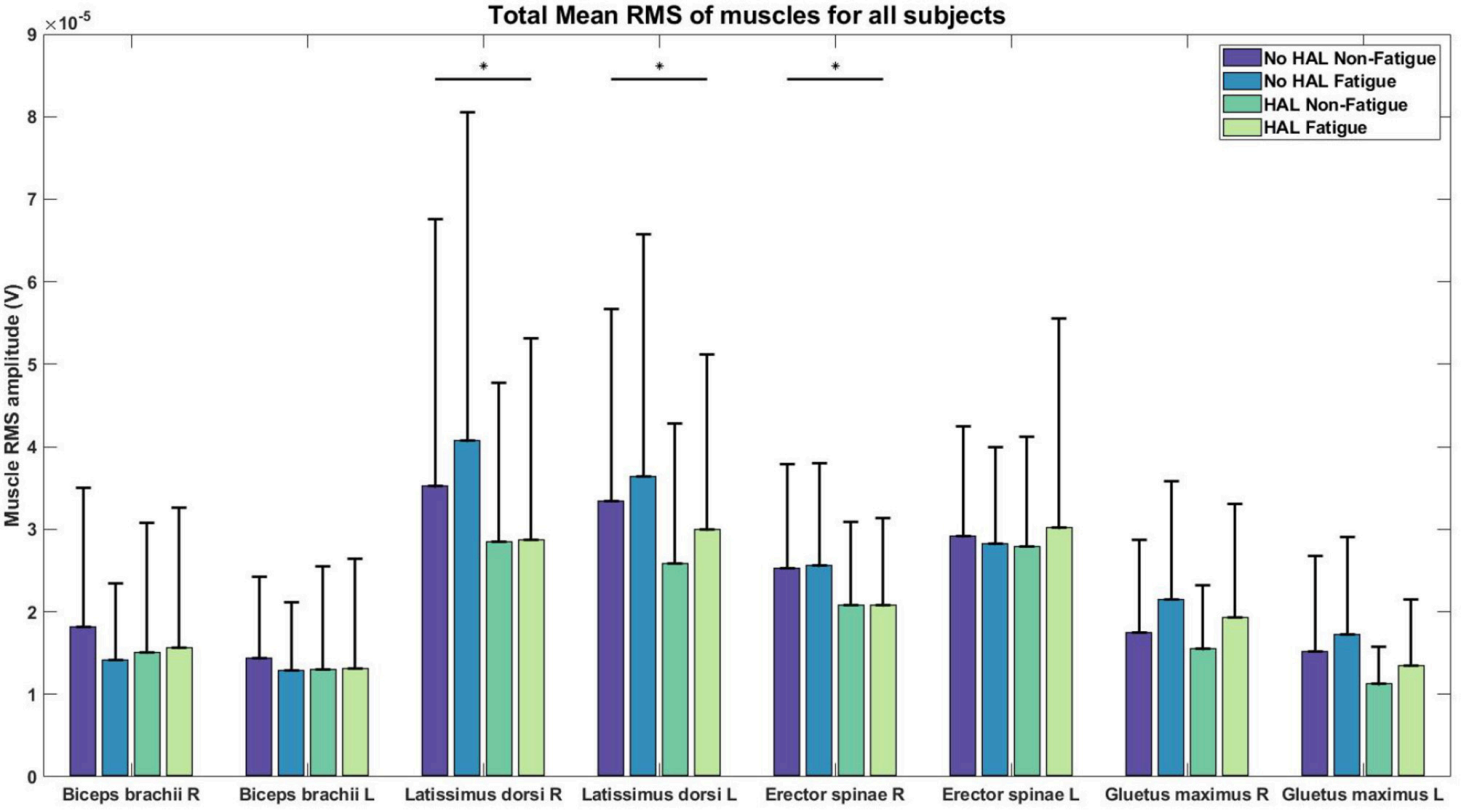
RMS values of EMG for each condition. Asterisks denote significance level of *p* < 0.05 for conditions involving HAL.

Figure 8 depicts the difference in RMS values in a graphical form while Table 1 provides a detailed view of the independent ANOVA comparisons between conditions. The RMS values are reported in the order of

1. No HAL Non-Fatigue
2. No HAL Fatigue
3. HAL Non-Fatigue
4. HAL Fatigue

**Table 1:** *P*-values of 2-way ANOVA analysis of RMS values for comparison between conditions.

** 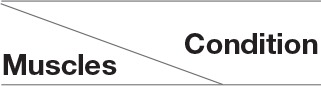 **	**HAL**	**Fatigue**	**HAL and Fatigue**
BB Right	0.6002	0.1947	0.0586
BB Left	0.8117	0.3675	0.3172
LD Right	**0.0465**	0.2876	**0.0477**
LD Left	**0.0232**	0.1121	0.4223
ES Right	**0.0353**	0.9282	0.7894
ES Left	0.9113	0.7881	0.5426
GM Right	0.1538	**0.0140**	0.8872
GM Left	0.0528	**0.0211**	0.8808

*Values in bold indicate significance (p < 0.05), while underlined values indicate marginal significance (0.05 > p > 0.06)*.

There was a significant effect of the HAL on the RMS values of the Right LD [**(1.)**3.5206*e*−05 ± 3.2421*e*−05 V, **(2.)**4.0727*e*−05 ± 3.9793*e*−05 V, **(3.)**2.8510*e*−05 ± 1.9211*e*−05 V, **(4.)**2.8761*e*−05 ± 2.4337*e*−05 V] and Left LD [**(1.)**3.3420*e*−05 ± 2.3219*e*−05 V, **(2.)**3.6415*e*−05 ± 2.9327*e*−05 V, **(3.)**2.5879*e*−05 ± 1.6962*e*−05 V, **(4.)**2.9967*e*−05 ± 2.1204*e*−05 V] muscles, as well as, in the Right ES [**(1.)**2.5311*e*−05 ± 1.2528*e*−05 V, **(2.)**2.5587*e*−05 ± 1.2384*e*−05 V, **(3.)**2.0858*e*−05 ± 1.0023*e*−05 V, **(4.)**2.0784*e*−05 ± 1.0576*e*−05 V] muscles. For non-HAL changes, fatigue significantly changes the RMS values of both the Right GM [**(1.)**1.7515*e*−05 ± 1.1252*e*−05 V, **(2.)**2.1528*e*−05 ± 1.4346*e*−05 V, **(3.)**1.5489*e*−05 ± 7.6860*e*−06 V, **(4.)**1.9371*e*−05 ± 1.3649*e*−05 V] and Left GM [**(1.)**1.5211*e*−05 ± 1.1590*e*−05 V, **(2.)**1.7249*e*−05 ± 1.1824*e*−05 V, **(3.)**1.1243*e*−05 ± 4.5360*e*−06 V, **(4.)**1.3490*e*−05 ± 7.9955*e*−06 V] muscles, although marginal significance was observed for the Left GM muscles when HAL was used (Table 1).

### 3.3. Number of Muscle Synergies and Reconstruction

**Figure 9:**
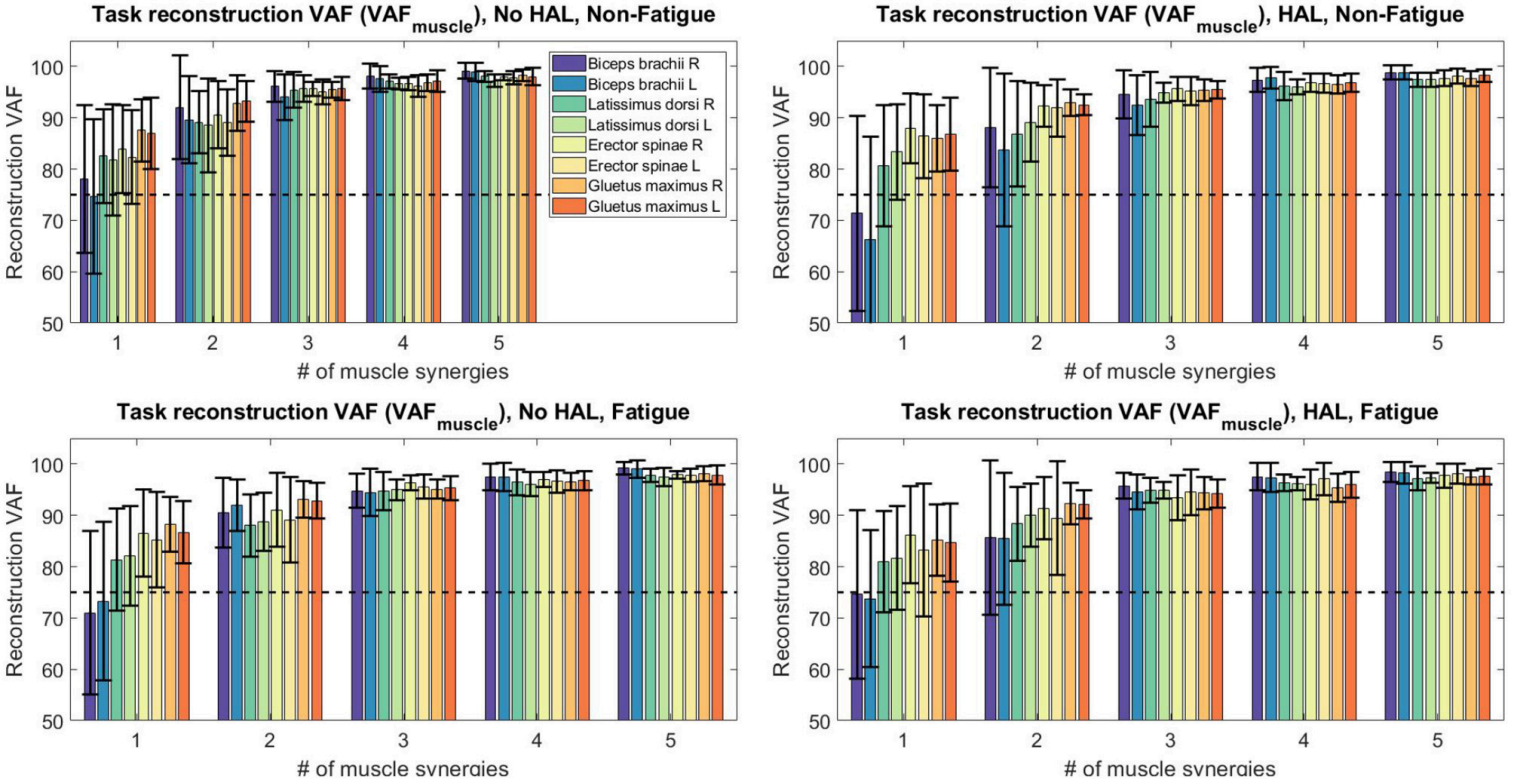
Reconstruction VAF values for all sessions and conditions. With 3 synergies, reconstruction quality of all subjects are above 75%.

Figure 9 above depicts the reconstruction VAF in relation to the number of synergies used for reconstruction. With the threshold set at 75% (dotted lines in Figure 9), we can see that 3 synergies are able to reconstruct the EMG profiles under different conditions sufficiently for all muscle vectors.

For further analysis, we fixed the number of synergies to 3, as it would provide the best trade-off between reconstruction quality and number of synergies. All subjects were used for further analysis.

### 3.4. Synergy Changes During Exoskeleton Use

**Figure 10:**
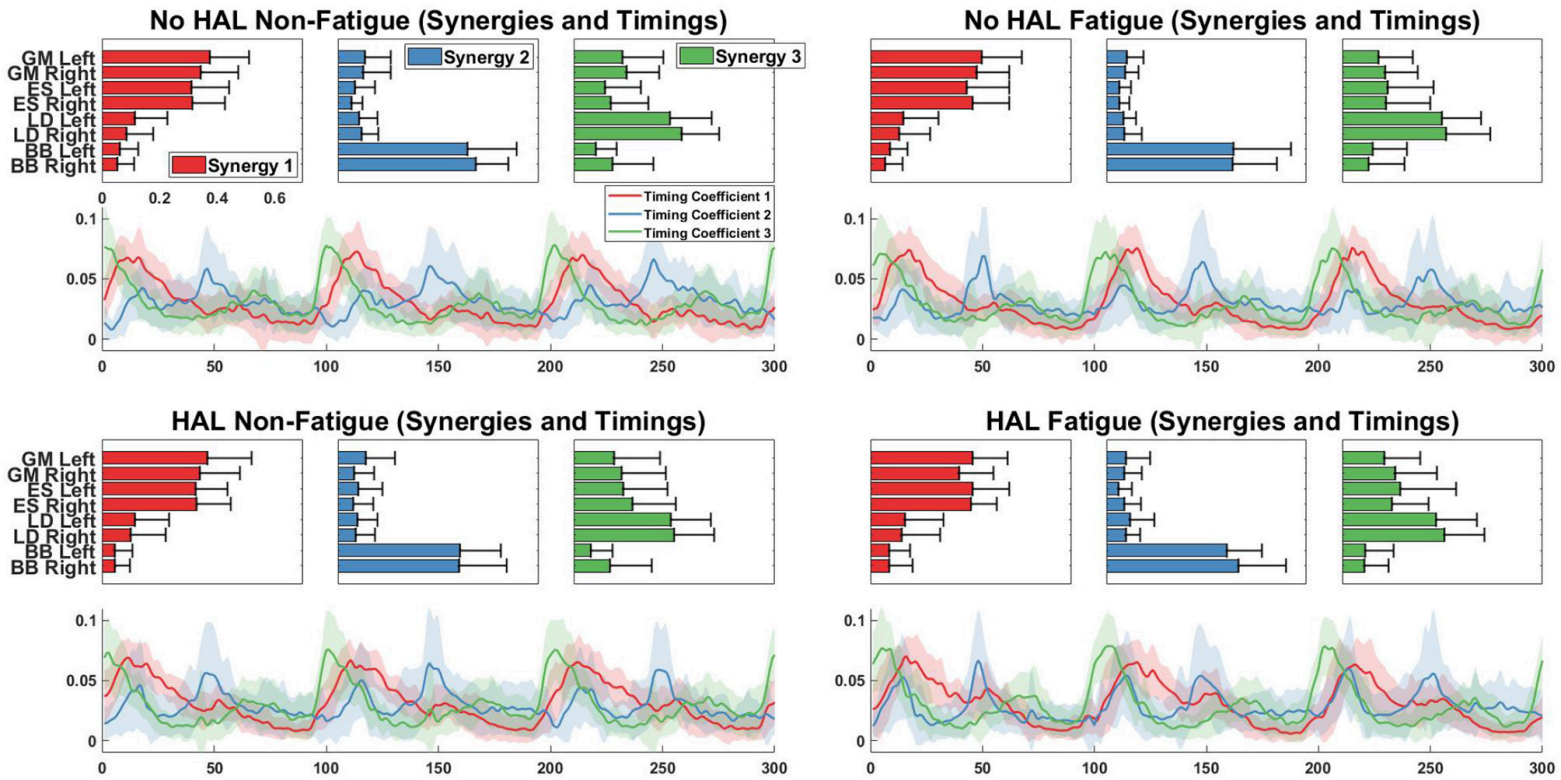
Synergy vectors and timing coefficients from all conditions. Each triple subplot depicts the mean of each synergy vector across all subjects. Error bars indicate the standard deviation. Timing coefficients are depicted below synergies. The bold lines indicate the mean timing coefficients across all subjects while the shading indicates the standard deviation.

Figure 10 shows the extracted synergies and timing coefficients from different conditions for all subjects.

**Figure 11:**
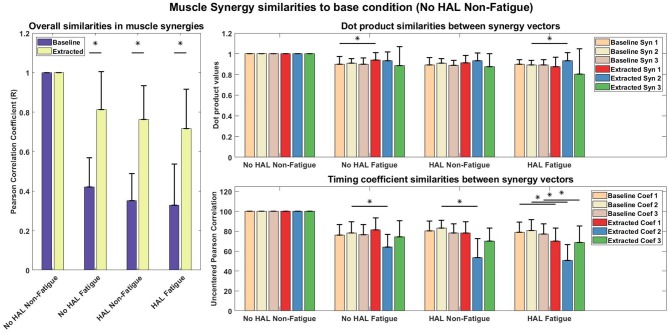
Muscle synergy and timing coefficient comparisons. The Left plot compares overall difference of sorted muscle synergy vectors from all conditions with the base condition, which is the No HAL Non-Fatigue condition. The Top Right plot depicts the dot product differences for each muscle synergy vector against another muscle synergy vector with the same position in the baseline condition. Similarly, the Bottom Right plot compares each timing coefficient vector with the timing coefficient vectors in the same position of the baseline condition. Asterisks denote significance of *p* < 0.05 of the Wilcoxon Signed-Rank test. “Baseline” indicate that the synergies were from randomly shuffled data, while “Extracted” refer to synergies extracted from actual data.

Figure 11 shows the comparison of synergies from different conditions with a chosen base condition, which is the No HAL Non-Fatigue condition. The overall difference of muscle synergy vectors between conditions extracted from subjects were significantly smaller than synergies extracted from the randomly shuffled EMG data. This is denoted by the higher similarity score of the extracted synergies, where (*No HAL Fatigue vs. No HAL Non-Fatigue:* 0.42 ± 0.15 vs. 0.81 ± 0.19, *p* = 0.00014 < 0.05), (*HAL Non-Fatigue vs. No HAL Non-Fatigue:* 0.35 ± 0.13 vs. 0.76 ± 0.17, *p* = 0.00014 < 0.05) and (*HAL Fatigue vs. No HAL Non-Fatigue:* 0.33 ± 0.21 vs. 0.72 ± 0.20, *p* = 0.000088 < 0.05).

A detailed look on the difference in muscle synergy contents with the scalar dot product showed that vectors in the 1st position of the No HAL Fatigue condition were significantly higher as compared to the baseline (0.90 ± 0.073 vs. 0.94 ± 0.072, *p* = 0.037 < 0.05). Also, synergy vectors in the 2nd position of the HAL Fatigue condition were significantly more similar as compared to the baseline conditions (0.89 ± 0.045 vs. 0.93 ± 0.077, *p* = 0.0072 < 0.05) (Top Right).

In the No HAL Fatigue condition, timing coefficients in the 2nd position were significantly different than the baseline (78.32 ± 11.27 vs. 63.92 ± 12.96, *p* = 0.0045 < 0.05). For the HAL Non-Fatigue condition, timing coefficients in the 2nd position were also significantly different than the baseline (83.04 ± 7.80 vs. 53.42 ± 18.99, *p* = 0.00012 < 0.05). Finally, in the last condition, all timing coefficients were significantly different to the baseline (1st position : 79.08 ± 10.19 vs. 70.14 ± 13.04, *p* = 0.014 < 0.05, 2nd position : 80.78 ± 11.02 vs. 50.66 ± 15.99, *p* = 0.000089 < 0.05, 3rd position : 77.26 ± 10.17 vs. 68.70 ± 16.46, *p* = 0.04 < 0.05).

**Figure 12:**
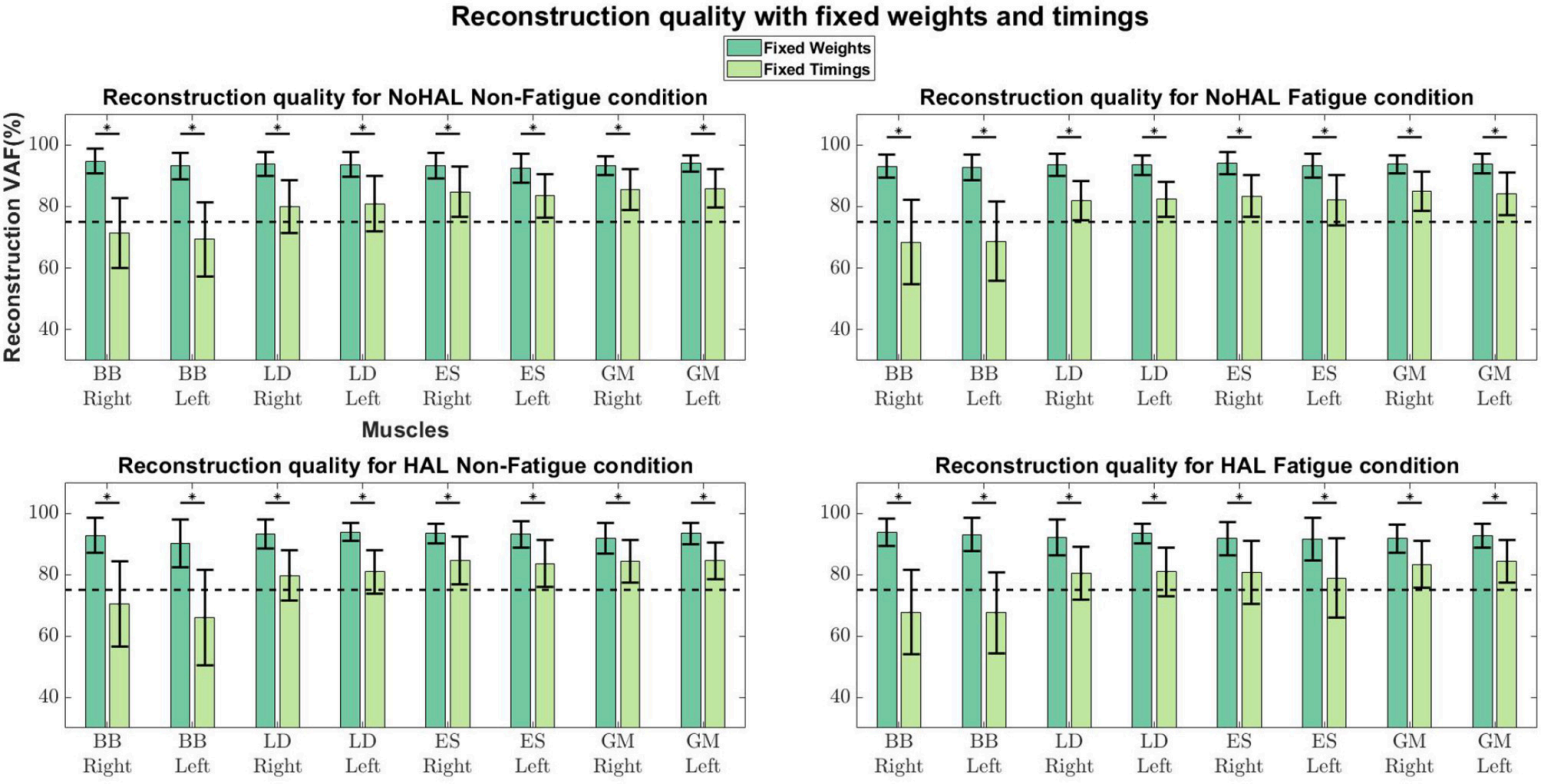
Synergy weights and timing coefficients were swapped between conditions. The bars on the left for each muscle shows the mean reconstruction quality (with the Uncentered Pearson Correlation Coefficient) for synergy weights from conditions different from the one being evaluated (e.g., For the No HAL Non-Fatigue condition, only reconstruction quality from the No HAL Fatigue, HAL Non-Fatigue and HAL Fatigue conditions were summed and compared). The right bars shows the reconstruction quality for timing coefficients different from the conditions as the synergy weights. Asterisks denote significance of *p* < 0.05 of the Wilcoxon Signed-Rank test.

**Figure 13:**
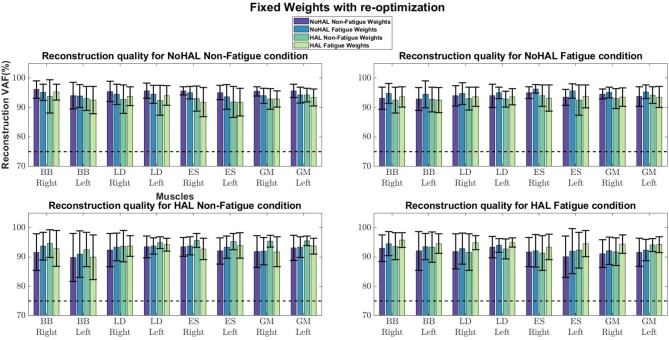
Synergies held fixed while timing coefficients were optimized.

**Figure 14:**
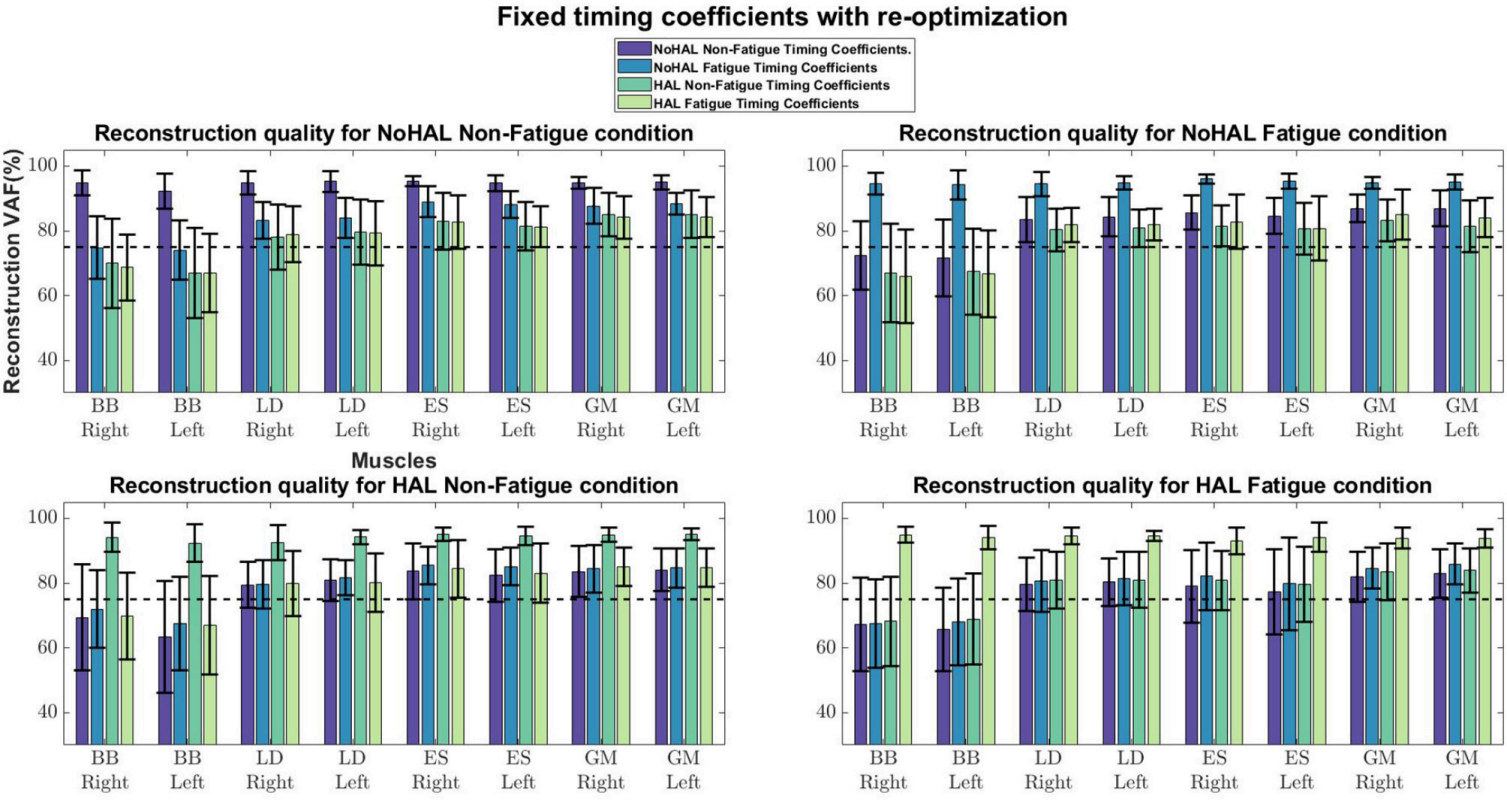
Timing coefficients held fixed while synergies were optimized.

Comparisons of reconstruction quality with fixed weights and timings (Figure 12) showed that when synergy weights were held fixed while the timings are free to vary, synergy weights gave a better reconstruction quality (values well above the 75% threshold), as opposed to the condition where the timings were fixed. A closer look at reconstruction qualities (Figures 13, 14) indicated that the reconstruction quality for both the Right and Left biceps were consistently poor, when the timings from different conditions were held fixed.

## 4. Discussions and Conclusions

Our current study aims to examine the effects of a lumbar support exoskeleton from the perspective of muscle coordination with muscle synergy analysis. For our experimental protocol, we assumed a fixed spatial set of muscle synergy weights, but variable recruitment (timing coefficients) for each condition. Chvatal and Ting (2012) provided evidence in their results and cited a multitude of studies that strongly support the assumption that modifications in human walking can be attributed to variances in the recruitment of spatially-fixed muscle synergies.

Our results indicate that muscle coordination patterns are significantly changed, mainly in the timing coefficients of the synergies and marginally changed in synergy weights, when subjects are using an exoskeleton. This change can be attributed to the assistance generated by the exoskeleton, as the dynamics of the movement is changed. Since the HAL Lumbar support exoskeleton is activated with muscle activity in the erector spinae, subjects would have to adjust their coordination to activate the exoskeleton at a pace that is comfortable for them. Results also indicated that muscles which were not supported by the exoskeleton, but relevant to the task, significantly change their outputs when the exoskeleton was used. This suggests that the central nervous system might be modulating muscle coordination in the entire body, instead of just muscles controlling the affected joint, by modulating the recruitment of muscle synergies.

In terms of task metrics, our results show that the HAL Lumbar support exoskeleton is able to assist subjects by increasing the number of lifting cycles a subject can perform. In addition, the perceived fatigue is also significantly lesser as compared to when the exoskeleton is not used. Further analysis with hip kinematics show that although hip angles in the upright posture is not significantly changed, regardless of conditions, hip angles in the stoop posture is significantly lower when the exoskeleton is used, but not between Non-Fatigue and Fatigue conditions (Figure 6, Bottom Left). This could be due to the fitting of the exoskeleton on subjects, causing a consistently lower hip angle in the stoop posture. Analysis of the angular velocities show that as subjects fatigue, angular velocities consistently decreased during transition from the stoop to upright posture (Figure 6, Top Right) and from the upright to stoop posture (Figure 6, Bottom Right). This occurred regardless of the use of the exoskeleton. This is because the exoskeleton is controlled with muscle activations of the subjects, and thus is able to scale its output according to the change in subjects' muscle activation levels. A detailed look at the kinematics change over time showed that subjects bent over more when using the HAL when starting the lifting action (Figure 7, Top Left) and at the end of the cycle, when placing the box down from an upright position (Figure 7, Bottom Left). Velocity profiles showed that in general, subjects move faster when using the HAL during a lifting action, when non-fatigued. Using the HAL when fatigued gave a similar velocity profiles as both Non-Fatigue and Fatigue conditions without HAL. However, the large standard deviation indicate that not all subjects exhibit this increase in instantaneous velocity.

Significant reduction in EMG amplitudes for the Right LD, Left LD, Right ES (Figure 8, Table 1) suggest that these muscles are assisted by the torque generated by the exoskeleton. This reduction in EMG amplitudes agree with results from previous studies with passive exoskeletons Lotz et al. (2009) and Bosch et al. (2016), who both reported reduction in erector spinae muscle activity and perceived muscular fatigue, together with an increase in muscle endurance. A recent study (Huysamen et al., 2018) evaluated an active exoskeleton for industrial use and also reported a reduction in erector spinae muscle activity. One notable result was the reduction of EMG activity in the LD muscles, as these muscles were not directly supported by the lumbar exoskeleton. A possible explanation could be that the corset-like design of the lumbar exoskeleton restricts movement in the lumbar vertebrae, allowing the assistive torque to be transmitted to the upper back, hence, reducing the load on the LD muscles. However, this would require further studies for verification.

Muscle synergy analysis with NNMF also showed a significant difference in the way subjects coordinate their muscles when using the exoskeleton. Our results in Figure 11 suggest that timing coefficients differences were dominating between conditions. This can be seen from the similarity value of the 2nd timing coefficient (53.42 ± 18.99) in the HAL Non-Fatigue condition. Timing coefficients were substantially different in the HAL Fatigue condition, where the similarity values of all timing coefficients were significantly lower than the chance level comparison (1st position : 79.08 ± 10.19 vs. 70.14 ± 13.04, 2nd position : 80.78 ± 11.02 vs. 50.66 ± 15.99, 3rd position: 77.26 ± 10.17 vs. 68.70 ± 16.46), suggesting that subjects might rely more on the lumbar support exoskeleton when they are tired. Other notable differences were the similarities between synergies extracted from the Non-Fatigue condition, with and without HAL (0.76 ± 0.17, Figure 11, Left), suggesting that muscle synergy weights were changed when the lumbar exoskeleton was used. This difference became larger in the Fatigue condition, as quantified by a lower similarity score than the Non-Fatigue condition. (0.72 ± 0.20, Figure 11, Left), suggesting greater modulation of the contents of the muscle synergies during fatigue. However, both were above the baseline comparisons for the HAL Non-fatigue and Fatigue conditions (0.35 ± 0.13 and 0.33 ± 0.21, respectively), which suggest that muscle synergy change might not be affecting the lifting motion. Still, there appear to be a decreasing trend in similarity values from Figure 11, (Left) and this could be investigated in a future work. Comparisons between each synergy vector indicate some minor modulation of the contents in the synergy vectors (Figure 11, Top Right), but they were above the chance level comparison values.

A related study examined muscle synergy change in walking with an ankle exoskeleton (Jacobs et al., 2018). They fixed synergy weights and timing coefficients between conditions (with and without the exoskeleton) to evaluate whether synergies and timings in one condition can be used to reconstruct EMG profiles in other conditions. They found that the weights and timings were able to reconstruct EMG profiles from other conditions better than random chance. However, differences in weights and timings extracted from different conditions were noted. (Jacobs et al., 2018) also noted that synergy weights gave better reconstruction quality, as compared to the timing coefficients, suggesting that changes in variability of EMG profiles were dominated by the activation of synergy weights.

Our results showed a similar trend, where synergy timing changes were dominating across conditions (Figure 11). Also, reconstruction quality with synergy weights were better than timings for all muscles (Figure 12), suggesting that timing coefficients were more important for good reconstruction of the EMG profiles. A closer look at Figure 14 showed that the biceps were having worse reconstruction quality than all the other muscles, indicating that the timings for activation of the biceps were varying across all conditions. This could be that the biceps were not directly supported by the lumbar exoskeleton and is activating independently. However, the relation of the muscles in the limbs and trunk are still unclear and would require further studies for verification.

### 4.1. Limitations of Study

One limitation of the study is that the gain parameter to activate and modulate the exoskeleton's output is not recorded. We manually adjusted the parameter until subjects feel comfortable controlling the exoskeleton. Future work can look into the relation of the gain parameter and the magnitude of change in muscle synergies. Another limitation is that upper limb kinematics were not captured in this study, which makes it difficult to explain changes in the upper limbs. Full body joint angle tracking could be a future consideration for further analysis. The age range of subjects in the study is also a limitation in this study as there might be contributions from age-related differences in reported fatigue. Age could be considered for a future study.

Another limitation may be that the number of measured muscles was relatively small. While Steele et al. (2013) showed that the selection of number of muscles impact the analysis of muscle synergies, one interpretation of their results is that the number of muscles are similar to a form of resolution. In Figure 5 of Steele et al. (2013), approximate logarithmic increasing similarity can be observed between subsets of selected muscles with the master set, as the number of muscle channels in each subset increase. This could indicate that there is a diminishing return effect with the increase in muscle channels. Having a higher resolution might not be useful for our task, since it is a relatively simple task. Steele et al. (2013) also showed that selecting large muscles can provide relatively high accuracy even with a small number of muscles. Since the larger muscles relevant to the motion were included in the analysis of this study, the measured muscles were considered to provide sufficient accuracy for our needs.

## Ethics Statement

This study was carried out in accordance with the recommendations of the University Guidelines for Clinical Trials, Institutional Review Board of University of Tsukuba Hospital, with written informed consent from all subjects. All subjects gave written informed consent in accordance with the Declaration of Helsinki. The protocol was approved by the Institutional Review Board of University of Tsukuba Hospital. The University Guidelines for Clinical Trials conforms to the ethical principles of the Declaration of Helsinki.

## Author Contributions

CT and HK collected, analyzed and interpreted the data. CT drafted the manuscript. KM, TA, MK, and MY planned and organized the experiments and provided ideas regarding lumbar load, fatigue and pain. YS originally developed HAL for lumbar support using bioelectric signals. KS designed the analysis and provided essential insight for the paper.

### Conflict of Interest Statement

YS is the C.E.O., shareholder, and director of CYBERDYNE Inc. which produces the robot suit HAL. CYBERDYNE was not involved in study design, data collection, analysis, writing or submission of this article. The remaining authors declare that the research was conducted in the absence of any commercial or financial relationships that could be construed as a potential conflict of interest.
